# Pain talk in hospice care: a conversation analysis

**DOI:** 10.1186/s12904-020-00625-x

**Published:** 2020-08-03

**Authors:** Yijin Wu

**Affiliations:** School of Translation Studies/Center for Medical Humanities in the Developing World, Qufu Normal University, No.5, Yantai Road, Donggang District, Rizhao, 250100 Shandong China

**Keywords:** Hospice care, Pain talk, Interactional resources, Conversation analysis

## Abstract

**Background:**

A large number of the hospice patients have been reported to be with symptoms of pain. Thus, managing the patient’s pain is one aspect of hospice care provision. The delivery of pain care services could be facilitated through effective communication. However, little has been done to explore the interactional details of the delivery of pain care services in palliative care.

**Methods:**

Conversation analysis is a useful method to explore the interactional details of interaction by hospice care providers and terminally ill patients. Using the method of Conversation Analysis (CA), this study aims to demonstrate how the hospice care provider employs different types of interactional practices to address the patient’s pain concerns. The data showed in this study are collected from the Alexander St website http://ctiv.alexanderstreet.com, an educational resource presenting a large collection of psycho-therapeutic videos.

**Results:**

In this study, an illustrative analysis is demonstrated to show the potential of conversation analysis for research on pain talk in palliative care. It has been shown that conversation analysis could contribute to unfolding the interactional details regarding “pain talk” in hospice care settings. Specifically, conversation analysis could provide a detailed description and interpretation of the conversational practices, which are used to construct hospice care provider participation in delivering pain talk. In addition, conversation analysis could also demonstrate the interactional resources by which patients disclose their experiences of physical or spiritual pain to the hospice care provider and the way how the hospice care provider responds to the patient’s troubles talk or feelings talk.

**Conclusions:**

This study identifies five types of interactional resources which are used to deal with the patient’s pain concerns in hospice care setting. A conversation analytical study of pain talk in hospice care could provide a turn-by-turn description of how the hospice care provider communicates with the terminally ill patient in terms of the patient’s pain concerns. The findings in this study could inform how the hospice care provider initiates, delivers and develops a pain talk with the terminally ill patient effectively.

## Background

Hospice care is a holistic and interdisciplinary approach to improving the quality of life of terminally ill patients and their families [1, 2]. Healthcare providers in hospice care work to relieve the physical, psychological, social, cultural and spiritual concerns of patients with life-limiting illness [3]. One of the key elements of physician-patient communication in any type of medical consultation (e.g., hospice care, acute care, primary care) is the solicitation of information from patients about their immediate concerns [4–8]. Using the method of conversation analysis (CA), a number of studies have been conducted to examine patients concerns in hospice care setting [9, 10]. Asking for information from patients about their concerns could contribute to a good understanding of their emotional, physical and psychological state in hospice care [1]. The type and amount of the patient’s concerns the hospice care provider could obtain has a strong influence on the quality of care received by the patient [11]. If a hospice care provider obtains only minimal information about a patient’s concerns, it is likely that the hospice care provider will not make an effective patient care plan [12]. Although CA is a very useful method for examining the patient’s concerns in hospice care [9–11], few studies has examined the ways in which the hospice care provider addresses the patient’s pain concerns in hospice care.

Pain is an important problem and primary concern of patients in hospice care [3]. 76–90% of hospice patients have been reported to be with symptoms of pain [13]. Hospice patients suffered from physical, psychological and spiritual pain [14]. Thus, managing the patient’s pain is one aspect of the palliative care provision. Hospice care providers should have good communication skills in managing the patient’s pain [15]. Hospice care providers with good communication strategies could address the patient’s psychological or spiritual pain effectively [14]. Pain management in a good communicative way could ease patient anxiety and fear and promote adherence to medication regimens [13]. Using the method of CA, this study provides a detailed description and interpretation of the conversational practices, which are used to construct the hospice care provider participation in delivering pain talk.

## Methods

### Patient and public involvement

The transcribed data showed in this study was obtained from two sessions of doctor-patient interaction in hospice care setting. The recorded data and its initial transcript were collected from the Alexander St website http://ctiv.alexanderstreet.com, an educational resource presenting a large collection of psycho-therapeutic videos. Since the 1970s, an increasing number of professionals have been consistently recording and transcribing their interactions with patients, which provides a great opportunity for the general public to understand the therapeutic process. This expanding archive of data has been published online by Alexander Street Press (www.alexanderstreet.com). This database is an online collection available to academic, public, and school libraries worldwide via annual subscription or one-time purchase of perpetual rights. The content covers a vast range of client issues such as addiction, autism, depression, grief, and palliative care. The initial transcript of hospice care provider-patient interaction could be seen at the right side of each video. The data were re-transcribed using Jefferson’s system [16]. Jefferson’s (2004) transcription conventions are organized into the following five categories: (1) transcript layout; (2) timing and sequential positions in talk-in-interaction,which show how different sequential positions in interaction are related in time; (3) detailed characteristics of speech delivery, including volume, emphasis, pitch change and so on. (4) metacommentaty and uncertain hearings. (6) transcription of accompanying talk, which involves non-verbal behavior such as sobbing, laughing and crying. We could see the details of Jefferson’s system of conventions at the end of this paper.

In this study, the data consists of two hospice care sessions lasting 50 min. A hospice care provider and two terminally ill cancer patients are involved. Study into two hospice care sessions can, by no means, tell the whole picture of pain talk in hospice care setting but might motivate hospice care providers’ reflection on their communicative skills and shed some light on how to deliver pain talk in hospice care setting.

### Data analysis

CA is the study of‘talk-in-interaction’. The general aim of CA studies is to show how conversation is sequentially organised, turn-by-turn and action-by-action, and how this sequence organisation in interaction enables interlocutors to manage intersubjectivity and communicate with each other smoothly [17–19]. CA insists on the necessity of recordings of naturally occurring activities (e.g. opening a medical consultation or closing a telephone call) for a detailed analysis of their endogenous organization. Conversation analysts hold that social activities are achieved collaboratively through their sequential activities [17]. The aim of CA is to examine the sequence organization of naturally occurring talk, and the social actions it achieves [18].

Distribution of typical patterns of social interactions in a corpus is of little importance in CA because the “environments of possible relevant occurrence’ cannot be known [20]. A primary tool of CA is to examine single cases [21]. As the discipline has grown, more analyses are based on large databases [22] and interventions based on CA results are proving robust when analysed statistically [23]. Thus, CA can be be used to examine not only single cases, but also large data sets. Conversation analysts qualitatively examine recordings of naturally occurring interactions to unravel the interactional practices through which social actions (such as request, invitation or pain talk in this study) are constructed in moment-by-moment processes [24]. An conversation analysis of an naturally occurring talk include the following three steps [20, 24]. First, observation. Conversation analysis begins with observation. Researchers should listen to and watch video-taped data carefully; Second, identifying a candidate phenomenon. Anything that could contribute to the smooth development of talk-in-interaction may be considered a candidate phenomenon for conversation analysis. For instance, social actions like request, invitation, and question are important conversation sequences, which could direct the development of talk-in-interaction; Third, describing an identified phenomenon. After identifying a candidate phenomenon, we need to describe target phenomenon or even related phenomena in detail. For instance, we could describe the sequential organization of the target phenomenon and explore its turn-taking system.

CA offers a rigorous method to the study of interaction in health settings [25]. In CA studies in medical settings, a key focus is how the doctor’s turn arises from the patient’s previous turn and how the doctor’s turn controls the patient’s subsequent turns (or vice-versa). As is pointed out by Peräkylä et al. (2008), CA provides a new way for observing and understanding medical interaction [26]. CA unfolds the sequential organization in the medical consultation in detail and explicates the practices and patterns through which the consultation is performed [27]. In this study, using the method of CA, a single case of pain talk in hospice care will be examined in detail.

## Results

This study focuses mainly on the hospice care provider’s interactional practices which work to deal with the patient’s pain concerns. By examining the provider’s interactional practices as they unfold within sequences of talk, we could have a better understanding of how the hospice care provider initiates, develops and completes a hospice care proposal.

### Proposing hospice in an implicit way

Conversations about end-of-life decisions and the transition toward hospice care remain among the most challenging communication tasks for hospice care providers [28–30], as initiating hospice care decisions often entails addressing a patient’s impending death. Consequently, the ability to initiate timely conversations about end-of-life decisions is considered a fundamental skill for hospice care providers [31]. Extract 1 shows an instance where the hospice care provider proposes hospice care in an implicit way.

### Extract 1

01 D: I think we need to talk about (0.5) what’s been going on for the last few days. The fact

02 that you didn’t respond to the spinal taps (.) I wouldn’t want to put you through any more

03 spinal taps. There’s going to be a time (.) when we’re not going to be able to deal with the

04 pressure (.) with the [steroids.

05 P: [Okay.

06 D:We will be able to help with pain and in making you comfortable.

07 I’m worried that your disease is progressing quickly.

08 We’ve talked about (.) you know (.) hospice before.

09 And I think this is the time where we need to discuss a bit more about it.

10 P:Well (.)Mary and I have talked many times and my thought again is I’m not afraid to die, but

11 I’m afraid of all the suffering that goes beforehand. So we just-we’re trying to find out,

12 you know, when that is going to come to pass just so we can-we can say goodbye to each

13 other.

At the beginning of this extract, the hospice care provider tells the patient that her illness reaches a point where spinal taps is not useful for dealing with her ongoing symptom. In line 5, the patient responds with a minimal response *okay*, which indicates that the patient orients to diagnosis as within the hospice care provider’s domain of expertise and thus accept the provider’s diagnosis without any query. Immediately after the patient’s agreement token *okay* in line 5, the hospice care provider starts to talk about hospice care in lines 6–9, where he first says that they could be able to deal with the patient’s pain and make her feel comfortable and then reports on the degree of her illness, where hospice care needs to be considered. Notice that in line 6 an exclusive *we* was used at the beginning of an claim of the treatment of the patient’s pain, which indicates the doctor is identified with other hospice care providers to deal with the patient’s pain. This could reassure the patient. In line 7, the hospice care provider says that he worried that the disease is progressing quickly, which implies that the disease may be out of control and the patient is approaching the end of life. Based on what the hospice care provider has said in lines 6 and 7, it can be supposed the patient’s illness cannot be cured whereas something could be done to improve her quality of life. It paves the way for the upcoming hospice care proposal. In line 8, the hospice care provider initiates the topic “hospice”, which indicates there are no clinical methods to cure the patient’s disease but improve her quality of life. The direct delivery of “hospice” could make the patient feel sad. It can be seen that the delivery of hospice is delayed by the use of “we have talked about” and “you know”. First, inclusive “we” works to evoke a sense of commonality and rapport between the hospice care provider and the patient. Then, by saying “*you know*” and leaving idea (hospice) less filled out, the hospice care provider could distance himself from potentially face-threatening utterance and invite the patient to participate in the ongoing talk. The hospice care provider begins to propose hospice care to the patient in line 9, where he propose the hospice care in an implicit way. First, the provider’s claim is prefaced with epistemic marker “I think”, which can be seen as the provider’s cautiousness in making knowledge claims. Specifically, through downplaying his own source of knowledge, the provider tries to preserve the patient’s greater rights to decide whether hospice care should be put into practice. Second, talking about hospice care is quantified by an approximator “a bit more”, which can both leave adequate leeway to the patient and take the paint’s decision-making into consideration. In this sense, it indicates that the provider wants to involve the patient in the subsequent interaction, which is, hoping to get confirmation from the patient concerning the use of palliative care.

### Soliciting the patient’s goals on pain management

Discussing goals of care requires a unique combination of good communication skills that should be separated conceptually from talking about prognosis or delivering bad news [32]. Understanding the patient’s care goals in the context of a serious illness invites the patient to participate in designing a shared care plan [32]. It is thus significant to understand how to solicit the patient’s care goals and how the patient responds to the provider’s inquiry. Let’s see extract 2 where the hospice care provider asks for the patient’s goals on pain management and the patient reports on her detailed goals on pain management.

### Extract 2

01D: What should our goals be?

02P: My goals (.) I-I don’t-I want to be pain-free. I’ll be honest about that (.) I want to be

03 pain-free, I don’t like pain. I-I don’t-I can’t-I can tolerate emotional pain to a certain point,

04 but I cannot tolerate physical pain. I want to have some sort of pain-free living standard

05 where I can go on you know. And I want people to know that around me that are working

06 with me that this is (.) this is it from me. You know, this is where I am going, this where I'm,

07 and this is where-where I want to be. I don’t want to (.) people, I don’t want to fool people.

08 I know where I’m going. I’m- I’m in a-I’m in a dangerous situation. You know (.) I may not

09 wake up tomorrow. I may not wake up tomorrow. And my (0.5) and my husband knows that.

10 I know that. Well (.) all of the preparations are done. Everything is ready. And this is the

11 place for us to do it. This is where we get the support that we need.

In line 1, the hospice care provider asks the patient“what should our goals be”, soliciting the patient’s expectation of therapeutic goals. By saying “our goals” instead of “your goals”, both the hospice care provider and the patient are involved in the design of pain management. On the one hand, it suggests that the provider and the patient are going hand in hand to deal with the patient’s pain. On the other hand, it offers an opportunity for shared decision-making, which will spark patient participation in her own care. Here, the hospice care provider’s question is designed to communicate that therapeutic goals being solicited are uncertain or unknown to him, which by their nature call for a response [19]. In response to the provider’s new-concern question, the patient: (1) begins her answer with “my goals”, then cuts herself off after“my goals-“and finally presents her goal, “I … I don’t … I want to be pain-free” where self-repair occurs [33]; (2) offers a detailed description of his goals with emotional disclosures (i.e., I don’t like pain, in line 3; I cannot tolerate physical pain, in line 4); and (3) elaborates on her perception and attitude towards her own illness, (i.e., I’m in a dangerous situation, in line 8; everything is ready, in line 10). In this sense, the patient displays her orientation to the doctor’s “What are our goals”as a solicitation of her expectation of pain management in palliative care.

### Soliciting the patient’s presenting pain concerns

After visits are opened, physicians typically solicit patients’ presenting concerns with questions such as *How are your feelings today*? [34, 35]. These questions have an important role to play in maintaining the effective communication between physicians and patients because different question designs/formats (i.e., different wordings) can significantly influence and constrain patients’ answers. Extract 3 shows an instance of how the hospice care provider uses questions to solicit the patient’s presenting pain concerns .

### Extract 3

01 D: Being in bed as you are right now, sitting quite still, do you have any pain at all right now?

02 P: No, no.

03 D: Oh, no pain right now?

04 P: No.

05 D: So what you are saying is that when your sit in bed as you are, you are comfortable?

06 P: Yes.

07 D: If you get up and try to walk, then your legs are painful?

08 P: Yeah, painful yeah.

At the beginning of this extract, the hospice care provider’s question, “do you have any pain at all right now? ” solicits an updated evaluation of the patient’s current situation. Turn-terminal, temporal modification, “right now”, invites the patient to evaluate the current state of her condition relative to its previous state (presumably during the prior visit). Here, the provider’s question prefers a negative response because of the use of the negative polarity term“any...at all” [36]. In what follows, the patient’s response in a brief and immediate way is aligned to that preference. Notice that the patient’s response “no” was repeated twice, which is a report of improvement on her health status and thus demonstrates a positive evaluation of her physical heath condition. Subsequently, the hospice care provider’s question in line 1 was expressed in an alternative way in line 3, that is, in a negative declarative question, “no pain right now? ” Here, the question “no pain right now” is polarized in a negative direction favoring a “no” response. Again, the patient’s response in line 4 is both aligned to the polarity preference expressed in the question, and produced in preferred format, that is, the response is designed briefly and produced without significant delay. In line 5, the hospice care provider initiates a new question, which is prefaced with a “so” positioning it as building on the patient’s prior talk. Here, the provider attempts to reformulate what the patient has said in the preceding turns and confirm the current state of her health status. After getting the patient’s confirmatory response, the hospice care provider in line 7 raises a new question, which draws out an implication of what they have talked about in the preceding turns. Notice that in this extract, three close-ended questions are designed to solicit an evaluation of an ongoing physical-health condition. Closed-ended questions communicate that although the hospice care provider has some idea about the nature of patient’s concerns, he does not have primary authority (including knowledge) over the sate of the patient’s pain concerns [37]. In other words, the patient has primary access to, and knowledge of, her pain concerns.

### Displaying affiliation with the patient’s pain concerns

In palliative care, patients could create opportunities to be empathic by displaying their affectual stance towards their symptoms of pain. In response, the hospice care provider deals with the patient’s pain concerns through affiliative displays. Extract 4 is a case in point.

### Extract 4

01 P: If I can walk without pain, that will be something fine =

02 D: =Ye:ah

(1.0)

03 P: I have had pain there for-for-for 5 months now.

04 D: Hmm (.) Hmm. Well (.) that’s what we’re aiming for (.) get rid of the pain (.) and get you

05 walking.

06 P: Yeah.

At the beginning of this extract, it can be seen that the patient is afflicted by pain (“If I can walk without pain”, line 1) and she expects to get rid of pain (“that will be something fine”, line 1). The hospice care provider responds to this with an acknowledgement token “yeah”. The provider does not continue his turn, and a 1.0 silence ensues. After this, the patient says that she suffers pains for 5 months, which suggests the patient has been eager to get the pain away, and can also be heard to pursue a stronger response from the provider. Proposals of affiliation were usually made after the patient had put some effort into pursuing affiliation from the professional [38]. The provider responds with a twice-repeated acknowledgement token ‘hmm’, then with a particle “well”. Turn-initial *well* functions as an alert that the talk to follow will privilege the speaker’s perspectives, experiences or feelings [39]. After the particle “well”, the hospice care provider deals with the patient’s pain concerns through affiliative displays (“we’re aiming for (.) get rid of the pain (.) and get you walking.”, lines 4 and 5), which affiliates with the patient’s ongoing concerns.

### Alleviating the patient’s pain concerns

hospice care providers should listen to their patients’ pain concerns actively and deal with them patiently. Hospice care providers usually tell patients that they could manage their pain well [40]. For patients, they would find it reassuring to know that their pains can be managed well. Extract 5 is case in point.

### Extract 5

01 D:When you wake up like that, it’s because you are worried or because of pain, or you just.

02 wake up?

03 P:It-It-It could be pain but no, and I don’t worry. Well (.) of course, when here, you (0.5) you

04 don’t know how it’s going to end and there is always a bit of anxiety against the same for.

05 everybody, but it was the same at home, I wouldn’t sleep at night (.) it was my nightmare too.

06D: What do you mean you don’t know how it’s going to end?

07P: Am I going to suffer? How is it going to (.) Am I going to get into a coma? It’s all question.

08 that you, we don’t know, but there is always, you know (.) you-you ask yourself.

09D: What do you think will happen? What do you imagine might happen?

10 P: Well, to me cancer, it means it’s not curable and you suffer a lot.

11 D: You suffer a lot like from pain.

12 P: Yeah, Yeah (.) Maybe I’m wrong today (.) maybe they’ve got [medication.

13 D: [Yeah.

14 P: but to me, that’s the way it is.

15 D: What if I told you that we do have the means and we do have the medication to control pain.

16 in almost all cases, like almost a hundred percent.

27 P: Yeah.

At the beginning of this extract, the hospice care provider inquires the patient about why she wakes up while other patients sleep. In response, the patient in lines 3–5 reports that she cannot sleep at night because of both anxiety and pain. In lines 12 and 14, the patient says that she wakes up mainly because of pain while other patients who sleep may have gotten medication. In what follows, the hospice care provider in lines 15 and 16 switches to talking about non-problematic and positive issues in managing the patient’s pain, which is a common way to exit from troubles-telling sequences in ordinary conversation [16]. Notice that the hospice care provider uses extreme case formulation“in almost all cases, a hundred percent” to display an orientation to the fact that the patient’s pain could be definitely controlled. In this sense, the provider’s presentation of comforting utterances tend to formulate the patient’s pain both as common and as basically relivable and manageable.

## Discussion

This study demonstrates how the hospice care provider uses different types of interactional practices to address the patient’s pain concerns. It is a huge communication challenge for hospice care providers to deliver a hospice proposal to the terminally ill patient. Delivering the hospice proposal in a direct or explicit way may make patients feel bad. As suggested by the practice the provider employed in Extract 1, proposing hospice in an implicit way is a good way to make a hospice proposal more bearable. Also, Gramling and Gramling (2019) found that palliative care providers tended to deliver bad news with a range of communication strategies based on intensive conversation analysis on how palliative care providers deliver bad news to patients or their family members [41].

In Extract 2, soliciting the patient’s goals on pain management is used as as an interactional resource for decreasing the doctor’s deontic responsibility for pain management, which invites the patient to participate in pain management. Thus, it highlights the patient-centered care, which encourages patients to become equal partners in the decision-making based on their expertise on stheir own health [42].

Soliciting the patient’s presenting pain concerns is achieved through four closed-ended questions in Extract 3. Closed-ended question greatly limited patient participation in the medical interaction. The use of closed-ended questions in Extract 3 could display high provider control over the medical interaction and the patient’s response. In this sense, closed-ended questions in this extract could show that the care provider takes predominance over the conversational flow of the ongoing medical interaction and the patient is thus not entitled to enough conversational space to express her feelings talk or troubles telling.

Hospice care provider not only treat the patients’ physical symptoms, but also attend to their psychological, social and spiritual needs [13]. Hospice care providers should identify and perceive patients’ psychological, social and spiritual needs through their utterances. Hospice care provider should pay special attention to the negative emotion words or phrases in the patient’s utterances, which could show the patient’s feelings talk or troubles telling. Extract 4 is a good case in point. In this extract, after perceiving the patient’s negative emotions, the hospice care provider shows empathy with the patient through affective understanding of what she has said. This is in line with Ford et al.’s (2019) research finding [4]. By conversation analysis of palliative care provider-patient interactions, Ford et al., (2019) found that palliative care providers use empathic utterances to display that they understand patients’ emotional experiences of their illness.

Doctors have less epistemic rights to knowledge about patients’ experience of symptoms and concerns about their illness than patients. In contrast, patients have more epistemic rights to know about their lifeworld experiences and concerns about their illness than doctors. Extract 5 is a good case in point. It could be seen through the four open ended questions (a semi-open -ended question with three optional answers in line 1, an open-ended question in line 6, and two open-ended questions in line 9), which encourages the patient to report her experience of symptoms and concerns about her illness. After figuring out the patient’s experience of symptoms and pain concerns, the doctor reports that the patient’s pain could be definitely controlled, which could alleviate the patient’s pain concerns effectively.

A strength is that this study examined in detail recorded real-life interactions between a hospice care provider and two terminally ill patients. CA allowed us to examine how terminally ill patients deliver their pain concerns and how the hospice care provider addresses the patient’s pain concerns. It seems that CA is a useful approach for the analysis of pain talk in hospice care. Also, there are limitations to this study. One limitation is that this study only involves a hospice care provider and two terminal patients, and thus the findings of this study are based on the interactional practices of three participants. This necessitates caution in generalizing to other hospice care settings and demonstrates that further research is needed to establish whether the interactional practices found in this study are employed in other hospice care settings. In addition, this study only involves participants who could speak English and does not concern hospice care providers and terminally ill patients who speak other languages. Furthermore, CA does not concern the speaker’s intended meaning and internal motivation, which plays an important role in maintaining the smooth communication between speakers and hearers. Face-to-face interview could address these shortcomings. However, the researcher could not be able to interview participants in this study. Despite these limitations, it could be argued that the interactional practices identified in this study may be potentially transferable to other hospice care settings.

## Conclusion

In this study, an illustrative analysis is demonstrated to show the potential of conversation analysis for research on pain talk in hospice care. It has been shown that conversation analysis could contribute to unfolding interactional details regarding “pain talk” in hospice care setting [4]. Specifically, conversation analysis could provide a detailed description and interpretation of the conversational practices, which are used to construct hospice care provider participation in delivering pain talk. Using the method of conversation analysis, this study identified five types of interactional resources which are used to deal with the patient’s pain concerns in hospice care setting. Conversation analytical studies on pain talk in hospice care provides a turn-by-turn description of how the hospice care provider communicates with the patient in terms of pain concerns. In addition, by examining sequential features of pain talk in hospice care setting, we may get a better understanding of the interactional details in process of achieving palliative care. This study not only could enrich studies of hospice care but also may help to improve hospice care providers’ communication skills. This is a preliminary study of pain concerns in hospice care, and thorough investigations would be carried out in future studies.

## Data Availability

The transcribed data are available on reasonable request from the corresponding author.

## Abbreviations

CA: Conversation analysis
